# Catalytic Reduction of High Concentration Nitrate-Bearing Industrial Wastewater for Ammonium Recovery

**DOI:** 10.1021/acsestwater.4c00897

**Published:** 2025-03-20

**Authors:** Dydia Tanisha González, José Alberto Baeza, Luisa Calvo, Miguel Ángel Gilarranz

**Affiliations:** † Departamento de Ingeniería Química, C/Francisco Tomás y Valiente 7, Universidad Autónoma de Madrid, 28049 Madrid, Spain; ‡ Institute for Advanced Research in Chemical Sciences (IAdChem), 16722 Universidad Autónoma de Madrid, 28049 Madrid, Spain

**Keywords:** NO_3_^−^ reduction, ammonia, Pd−Cu catalysts, wastewater treatment, pH control

## Abstract

The concept of NH_4_^+^/NH_3_ production from wastewater treatment for subsequent recovery and reuse has gained much attention in the past few years. The conversion of NO_3_^–^ present in wastewater into NH_4_^+^/NH_3_ through catalytic reduction is a sustainable approach for resource utilization from wastewater. In the present work, the application of this technique to industrial wastewater with a high NO_3_^–^ concentration is proposed. A good activity of Pd–Cu catalysts supported on carbon black was observed at high NO_3_^–^ concentrations. Complete NO_3_^–^ conversion was achieved in water with an initial concentration of 1000 mg/L and a catalyst concentration of 0.93 g/L in a stirred reactor. Furthermore, an improvement in catalytic activity was obtained when the reactor was operated without pH control, resulting in an increase in NH_4_^+^ formation to 184 mg/L. Additionally, in successive 8-h reaction cycles with a reaction medium equivalent to that of industrial wastewater, a progressive decrease in catalytic activity was observed, which may be associated with a noticeable decrease in the Pd^0^/Pd^n+^ species ratio.

## Graphical abstract

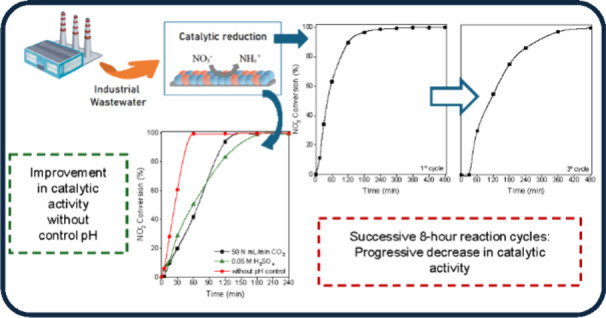

## 1 Introduction

NO_3_^–^ accumulation in water bodies has increased in recent decades due to pollution from different sources related to population growth, wastewater discharge, increased livestock farming, aquaculture, and the excessive use of fertilizers in agriculture.^1^ Water pollution by NO_3_^–^ can have numerous harmful effects, including eutrophication, commonly described as the nutrient enrichment of water. The increase in nutrient content leads to the proliferation of algal biomass, which causes secondary effects such as reduced light penetration and dissolved oxygen levels in the water, resulting in anoxic conditions and, consequently, the alteration of aquatic habitats.^2,3^

Wastewater with high NO_3_^–^ content is generated in several industrial processes, such as fertilizer and explosives production.^4^ The fertilizer industry generates effluents with diverse characteristics depending on the primary reagents used in production and their origins in the process streams. Generally, wastewater from a fertilizer production plant is rich in NO_3_^–^, NH_3_, acids, alcohols, and salts.^5^ On the other hand, wastewater from the explosives industry is mainly characterized by a low pH and high concentration of SO_4_^2–^, NH_4_^+^, NO_3_^–^, NO_2_^–^, and COD.^6^ Wastewater denitrification in the fertilizer industry is usually carried out through biological treatment or ion exchange.^5^ Technologies such as activated carbon adsorption, electrolysis, coagulation, and others are commonly used in the treatment of wastewater from the explosives industry.^6^ However, secondary polluted streams are generated during the treatment of wastewater from both industries using these technologies, and the integration of two or more physical, physicochemical, or biological methods may be necessary.^5,6^

A growing number of studies have identified the reduction of NO_3_^–^ in wastewater to NH_3_/NH_4_^+^ ^7^ as an alternative to the conventional Haber–Bosch process, offering a method to simultaneously remove and valorize NO_3_^–^. Significant attention has been paid to electroreduction methods;^8,9^ however, the feasibility of large-scale NH_3_ production remains questionable due to high energy costs. Likewise, while bioelectrochemical systems (BES) using microorganisms as biocatalysts have proven efficient in nearly complete NO_3_^–^ removal, further investigation into the electron transport pathway is needed to elucidate the mechanisms for the production of NH_4_^+^.^10^ Therefore, numerous works focus on the feasibility of nitrogen recovery in BES by treating wastewater with high NH_3_ concentration.^11^ In this process, total ammoniacal nitrogen (TAN) is recovered by shifting NH_4_^+^ to NH_3_ under alkaline conditions, followed by recovery through precipitation, stripping combined with absorption, or membrane-based extraction processes. Among these, stripping is currently the most widely used recovery process, as it offers lower energy consumption compared to conventional systems.^12^

Catalytic reduction is another interesting technique for removing NO_3_^–^ from water, producing mainly N_2_, NH_4_^+^, and NO_2_^–^. This technique has been extensively studied for NO_3_^–^ removal from polluted groundwater, where the NO_3_^–^ concentration is much lower than in industrial wastewater, aiming to produce drinking water by maximizing the selectivity to innocuous N_2_.^13^ This technique is less investigated for NH_4_^+^ production compared with other treatments, such as electroreduction. However, the knowledge acquired for N_2_ production can be applied to industrial wastewater treatment. Chemical reduction typically involves the use of a reducing agent, usually H_2_, and a bimetallic catalyst, with Pd or Pt as the noble metal, and Cu, Sn, or In as the promoter metal, being among the most used catalysts.^14,1^ The reaction mechanism of NO_3_^–^ reduction on a bimetallic catalyst has been described by several authors.^15–17^ In the first stage, NO_3_^–^ adsorbs onto the promoter metal sites, where it is reduced to aqueous NO_2_^–^. The promoter metal is then reduced again by the noble metal, which is activated by H_2_ adsorption and dissociation, thereby participating in a redox cycle. In the second stage, NO_2_^–^ adsorbs onto the monometallic noble metal sites and is reduced to other intermediates, such as NO. Depending on the evolution of this intermediate, either N_2_ or NH_4_^+^ is obtained as the final product. It has been shown that if NO is hydrogenated to species like HNO or NH, the formation of NH_4_^+^ is favored, as illustrated in Figure 1.^14^ The product distribution is strongly dependent on several factors, including the pH of the medium, the type and concentration of the reducing agent, the active phase, and the catalytic support, among others.^1^ Therefore, under appropriate conditions, the chemical reaction of NO_3_^–^ could be directed toward NH_4_^+^ production.

**Figure 1:**
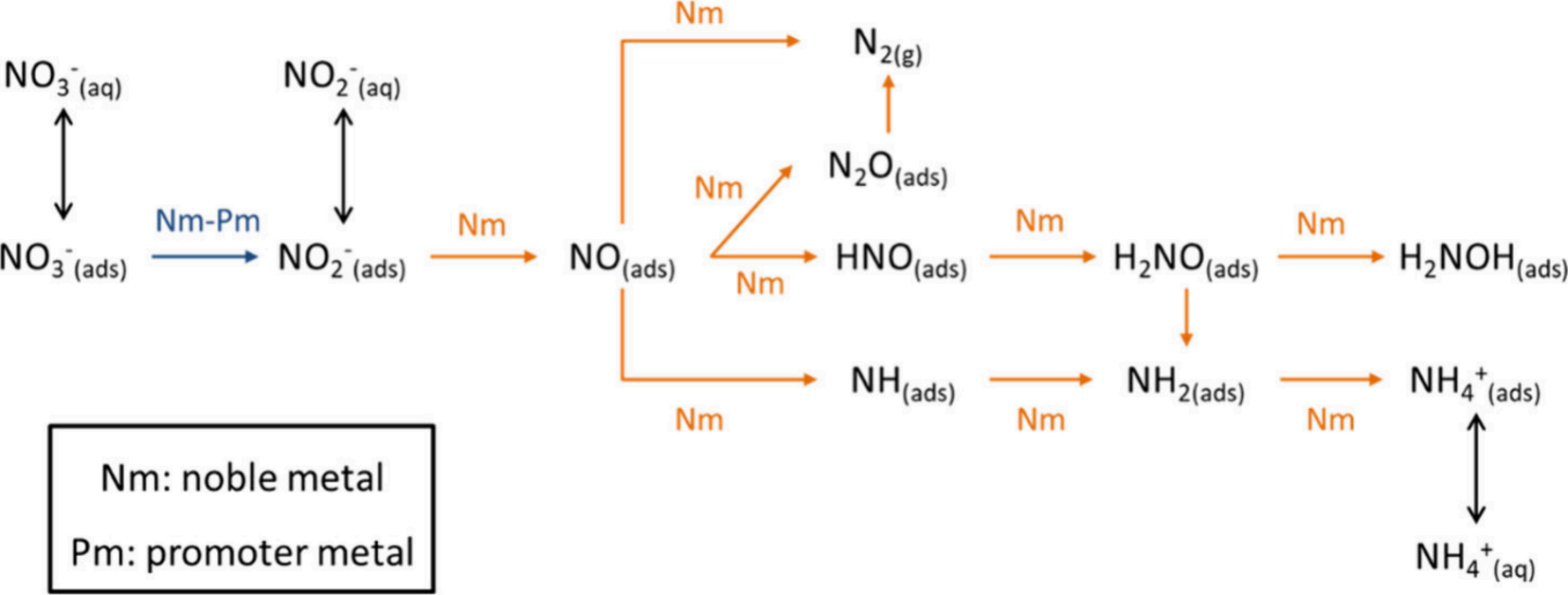
Mechanism NO_3_^–^ reduction over a bimetallic catalyst. Adapted from Martínez et al.^14^

To apply NO_3_^–^ chemical reduction as a technique for treating wastewater and recovering nitrogen as NH_4_^+^, the activity and stability of the catalyst in the presence of salts is crucial. In the application of chemical reduction for drinking water treatment, salts in the water matrix have been shown to influence the catalytic process, particularly HCO_3_^–^, which leads to lower activity in NO_3_^–^ chemical reduction due to competitive adsorption on the catalyst.^18^ In the presence of other anions, such as SO_4_^2–^ and Cl^–^, the negative effect of HCO_3_^–^ on the catalytic activity decreases.^19^ On the other hand, some works in the literature indicate that salts can cause catalyst deactivation due to poisoning of the metallic phase.^20,13^ However, in a previous work, we observed a high tolerance of Pd–Cu catalysts supported on carbon black to the salts present in commercial drinking waters, achieving total NO_3_^–^ conversion after 1 h of reaction.^18^

In the current work, catalytic reduction is proposed for the treatment of simulated fertilizer/explosive industry wastewater with a high NO_3_^–^ concentration, with the goal of producing NH_4_^+^. The knowledge gained from NO_3_^–^ reduction in drinking water is applied to maximize selectivity to NH_4_^+^, through variables such as the catalyst concentration and pH control. Likewise, catalyst stability is assessed in consecutive reaction cycles.

## 2 Experimental Section

### 2.1 Materials

PdCl_2_ (>99.9%), CuCl_2_·2H_2_O (>99.9%), 2,6-Pyridinedicarboxylic acid (>99.5%), and CaCl_2_ (>99.5%) were provided by Sigma-Aldrich. NaNO_3_ (99%), NaNO_2_ (98%), NaNO_3_ (98%), (NH_4_)_2_SO_4_ (>99.0%), Na_2_CO_3_ (99%), NaHCO_3_ (99.5%), HNO_3_ (65%), and H_2_SO_4_ (95–98%) were supplied by Panreac. H_2_ (>99.999%) and CO_2_ (>99.99%) were supplied by Nippon gases. Carbon black ENSACO350G (ENS350) was purchased from Timcal.

### 2.2 Catalyst Preparation and Characterization

The bimetallic Pd–Cu catalyst was prepared using a commercial ENS350 carbon black as a support with a 5 wt % nominal metal phase load and a Pd:Cu mass ratio of 2:1. Successive impregnation was used for the preparation of the catalysts since previous works have demonstrated superior performance of the catalysts in NO_3_^–^ reduction.^21^ A solution of PdCl_2_ in 0.1 M HCl was impregnated into 1 g of the support using a rotary evaporator at 70 °C, 200 rpm, and 150 mbar until dryness. In a second step, a solution of CuCl_2_·2H_2_O prepared in milli-Q water was impregnated following the same procedure indicated for Pd impregnation. After each impregnation step, the catalyst was dried in an oven at 60 °C for 24 h. It was then calcined at 200 °C for 2 h in an air atmosphere and finally reduced at 200 °C under a H_2_ flow for 1 h (25 N mL/min).

Fresh and used catalysts were characterized by X-ray Photoelectron Spectroscopy (XPS), using a K-Alpha Thermo Scientific instrument equipped with an AlKα X-ray excitation source, at 1486.68 eV. XPSPEAK version 4.1 software was used to determine the metal species on the catalyst surface. The deconvolution of the Pd peaks was performed considering that the Pd 3d 5/2 level shows two contributions, Pd^0^ in the 335.5–336.2 eV range and Pd^n+^ in the 336.8–337.7 eV range. Similarly, the Pd 3d 3/2 level showed two contributions in the 340.8–341.6 eV and 342.5–343.4 eV ranges for Pd^0^ and Pd^n+^, respectively. The contributions for Cu peaks are in the 932.0–932.9 eV range for Cu^0^ and 934.0–934.8 eV for Cu^n+^. Furthermore, these catalysts were characterized by Transmission Electron Microscopy (TEM), using JEOL JEM 2100 with EDS, Oxford instruments, and ImageJ software for the measurement of nanoparticle mean diameter. Elemental maps of the catalyst were obtained by energy-dispersive X-ray spectroscopy (EDX) coupled with a FEI Talos F200X microscope.

### 2.3 Catalyst Evaluation

The Pd–Cu/ENS350 catalyst was evaluated in 4 h tests conducted in a stirred batch reactor at 30 °C and atmospheric pressure (Figure S1 - Supporting Information). In general, the tests were performed with a total water volume of 150 mL, a catalyst concentration between 0.4 and 0.93 g/L, and under vigorous stirring. H_2_ was bubbled at 50 N mL/min as a reducing agent. Before starting the reaction, CO_2_ was bubbled at 50 N mL/min, or a 0.05 M H_2_SO_4_ solution was continuously added using a Metrohm 809 Tritando for pH control. Catalytic behavior was studied using NO_3_^–^ solutions in deionized water and two types of synthetic wastewaters (S1 and S2) with composition similar to those of the fertilizer and explosive production industries.^22,6^ Both solutions contained NO_3_^–^ and NH_4_^+^ at similar concentrations, while S2 had a higher total salt content (Table 1). Moreover, the catalyst stability was studied by reusing it in several consecutive runs. After each run, the final reaction suspension was vacuum filtered to recover the catalyst. The recovered catalyst was then dried for 24 h at 60 °C in an oven and used in the next reaction cycle under the same conditions. These tests were carried out for 8 h of reaction in 3 consecutive runs. Prior to the reaction experiments, adsorption tests on the catalysts were carried out in the absence of H_2_. In these tests the adsorption of NO_3_^–^ was found to be less than 5% (Figure S2-Supporting Information), therefore, disappearance of NO_3_^–^ in reaction tests can be ascribed essentially to conversion.

**Table 1:** Composition of the Synthetic Waters Tested

Water tested	NO_3_^–^ (mg/L)	NO_2_^–^ (mg/L)	Cl^–^ (mg/L)	SO_4_^2–^ (mg/L)	Na^+^ (mg/L)	NH_4_^+^ (mg/L)	Ca^2+^ (mg/L)
S1	947.1	—	—	289.2	351.1	108.3	—
S2	911.1	81.7	205.2	301.9	378.5	113.1	231.2

During the tests, liquid samples were taken at different reaction times to monitor the conversion of NO_3_^–^ (eq 1) and the concentrations of both NO_2_^–^ and NH_4_^+^. No measurements of the gas phase were made to determine the N_2_ concentration; therefore, a total N balance cannot be established. However, there is consensus in the literature indicating that in NO_3_^–^ reduction only N_2_ and NH_4_^+^ are generated as free final products and NO_2_^–^ as the intermediate free product.^14^ To better monitor the production of the desired product, the percentage of NH_4_^+^ production from the oxyanions converted was calculated for all reactions (eq 2). The results are shown in Table S1-Supporting Information. NO_3_^–^ conversion and NH_4_^+^ production were calculated as

$$NO_{3}^{-}\;conversion\;\left(\%\right)=\frac{n_{NO_{3}^{-},t=0}-n_{NO_{3}^{-},t}}{n_{NO_{3}^{-},t=0}}\cdot 100$$ 1

2 $$NH_{4}^{+}\;production\;\left(\%\right)=\frac{n_{NH_{4}^{+}final}}{n_{Nconverted}}\cdot 100$$

where $n_{NO_{3}^{-},t=0}$ represents the initial NO_3_^–^ concentration in mmol/L, $n_{NO_{3}^{-},t}$ is the NO_3_^–^ concentration in mmol/L at the time t (min), $n_{NH_{4}^{+}final}$ represents the NH_4_^+^ concentration at the end of reaction expressed in mmol/L, and *n*_*N converted*_ is the concentration of oxyanions converted in mmol/L.

The liquid samples were filtered using a PTFE filter with a pore size 0.45 μm and analyzed by ion chromatography (Metrohm 882 Compact IC plus anion and cation) to determine the concentration of NO_3_^–^, NO_2_^–^, NH_4_^+^, Na^+^, Ca^2+^, Cl^–^, and SO_4_^2–^. A Metrosep C6 column using a 1.7 mM HNO_3_^–^ + 1.7 mM 2,6-Pyridinedicarboxylic acid eluent (0.9 mL/min) was used for cation separation, and a Metrosep A Supp 5 column using a 3.2 mM Na_2_CO_3_ + 1 mM NaHCO_3_ eluent (0.7 mL/min) was used for anion separation. In addition, samples collected after 4 h of reaction were analyzed by total X-ray reflection (TXRF) using a Bruker S2 PicoFox TXRF spectrometer to determine the metal content.

## 3 Results and Discussion

### 3.1 Influence of NO_3_^–^ Initial Concentration

First, chemical reduction tests were carried out in water with different NO_3_^–^ concentrations (100–2000 mg/L). The pH was controlled using 50 N mL/min CO_2_ (Figure 2a) or a 0.05 M H_2_SO_4_ solution (Figure 2b). For experiments with NO_3_^–^ initial concentrations in the 100–500 mg/L range, conversion values higher than 99% were obtained after 4 h for both pH control agents. However, for initial NO_3_^–^ concentrations of 1000 and 2000 mg/L, much lower conversions were observed, with slower reaction occurring when the pH was controlled with CO_2_. The control of the pH in chemical reduction is crucial because NO_3_^–^ reduction produces OH^–^ ions, as described by eqs 3–5, which can block the active sites of the catalyst and affect catalytic activity.^15^ With CO_2_, the reaction medium’s pH is controlled by the carbonic acid - bicarbonate buffer formed, which is also influenced by the initial concentration of NO_3_^–^ and the extent of conversion achieved. In the runs depicted in Figure 2a, the pH ranged from 6 to 7, whereas with H_2_SO_4_, the pH remained between 5 and 5.5. Previous results on chemical reduction in mineral waters with NO_3_^–^ concentration of 100 mg/L showed similar activity of the Pd–Cu/ENS350 catalysts for both CO_2_ and H_2_SO_4_ pH control agents.^23^ However, the current experiments at high NO_3_^–^ concentration show that pH control with H_2_SO_4_ provides a pH range that leads to higher catalytic activity. Likewise with CO_2_, the concentration of HCO_3_^–^ increases as OH^–^ ions are released due to NO_3_^–^ and NO_2_^–^ reduction and subsequently neutralized. This accumulation of HCO_3_^–^ may also contribute to the lower activity observed, due to competition for adsorption at the promoter metal sites. Even at lower reaction times, particularly in the runs at an initial NO_3_^–^ concentration of 2000 mg/L, a slower reaction rate is noted. According to the NO_3_^–^ conversion results obtained, it seems that for higher NO_3_^–^ concentrations, it is more favorable to operate within a pH range similar to that achieved with H_2_SO_4_. These results are in good agreement with kinetic constants obtained from the fit to a pseudo-first-order kinetic model, as represented by the dashed lines in Figure 2 and summarized in Table S2-Supporting Information.

3 $$NO_{3}^{-}+H_{ads}\to N{O_{2}}_{ads}+OH^{-}$$

4 $$NO_{2}^{-}+H_{ads}\to NO_{ads}+OH^{-}$$

5 $$NH_{3}+H_{2}O\to NH_{4}^{+}+OH^{-}$$

**Figure 2:**
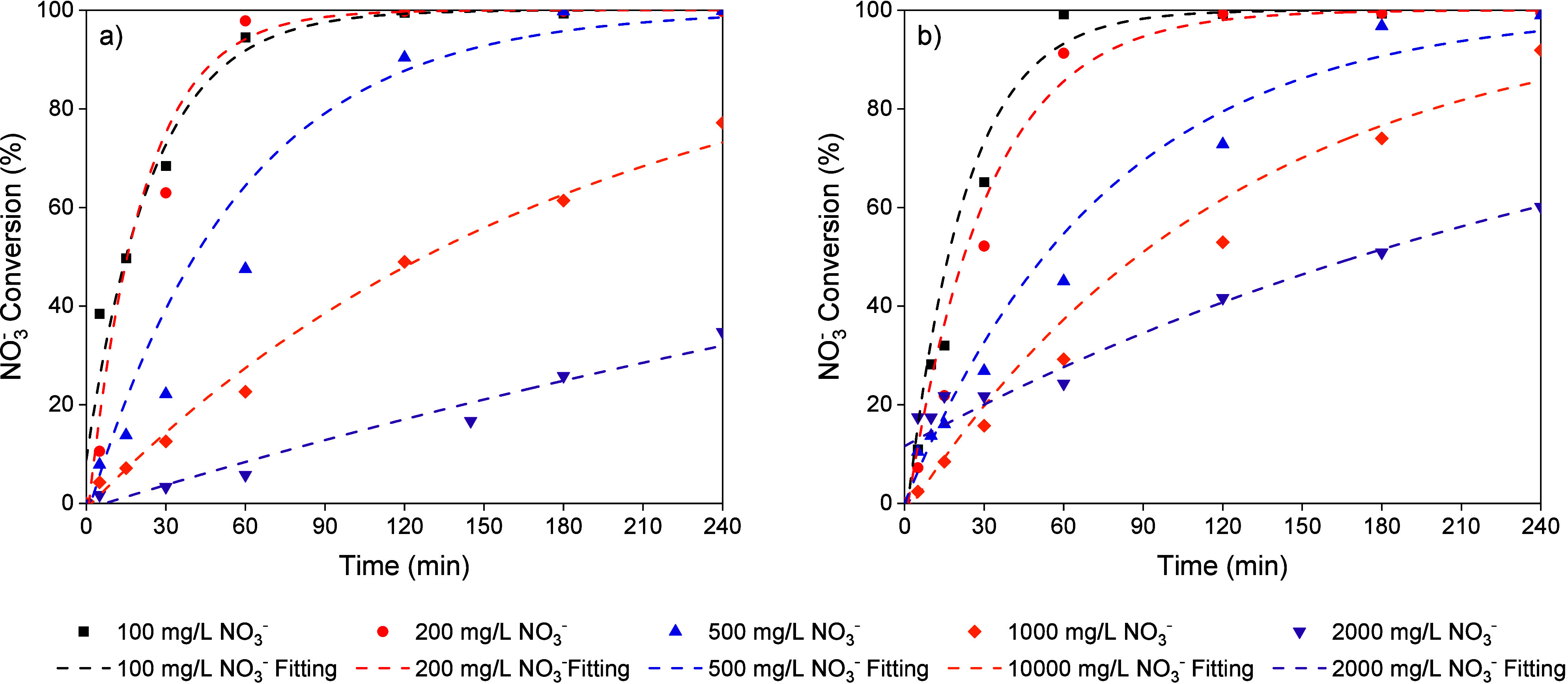
NO_3_^–^ conversion vs reaction time at different NO_3_^–^ initial concentrations using (a) CO_2_ (50 N mL/min) and (b) H_2_SO_4_ (0.05 M) as the pH control agent (H_2_ flow: 50 N mL/min, 0.4 g/L catalyst).

In Figure 3, the concentrations of NO_2_^–^ and NH_4_^+^ vs NO_3_^–^ conversion are depicted for the runs carried out at different initial NO_3_^–^ concentrations. In all runs, NO_2_^–^ was generated, reaching a maximum concentration when NO_3_^–^ conversion values were between 20 and 60%. The concentration of NO_2_^–^ was an order of magnitude lower in the reactions using CO_2_ as the pH control agent, with a peak concentration of ca. 4 mg/L at higher NO_3_^–^ concentrations. In contrast, with H_2_SO_4_ as the pH control agent, the NO_2_^–^ concentration peak exceeded 50 mg/L. Figure S3 (Supporting Information) plots the NO_2_^–^ concentration vs OH^–^ generated, calculated from the reduction of NO_3_^–^ and NO_2_^–^. It can be assumed that the neutralization of the OH^–^ ions generated leads to the incorporation of stoichiometric amounts of HCO_3_^–^ and SO_4_^2–^ into the reaction medium. A more negative effect of SO_4_^2–^ is observed, contrary to previous findings on the influence of salt presence in the reaction medium.^18^ The higher NO_2_^–^ accumulation in reactions with H_2_SO_4_ as the pH control agent indicates lower activity of Pd sites in the bimetallic catalyst, which may be related to the interaction of Pd and the SO_4_^2–^ ion added during the neutralization of OH^–^ ions. Moreno-Gonzalez et al.^24^ observed that SO_4_^2–^ ions strongly interact with Pd hydride (Pd–H) sites, potentially hindering NO_2_^–^ adsorption and its subsequent reduction. However, this interaction does not seem to affect the reduction of the Cu phase by the Pd pair in the catalyst as the activity for NO_3_^–^ reduction remains unaffected. Despite this, at longer reaction times and NO_3_^–^ conversions, NO_2_^–^ concentrations generally declined to well below 4 mg/L. Interestingly, it has been reported that active sites on the terraces of Pd nanoparticles are more selective to N_2_.^25^ Therefore, the higher NH_4_^+^ production in the run with an initial NO_3_^–^ concentration of 2000 mg/L could be linked to an increased interaction of the SO_4_^2–^ ion at these sites. Likewise, in works using catalysts with a high promoter metal to noble metal ratio, NO_3_^–^ is reduced at a high rate, promoting rapid NO_2_^–^ production that accumulates in the reaction medium due to the limited availability of noble metal active sites. This limits the pairing of N species and favors the hydrogenation of intermediates to form NH_4_^+^.^15^

**Figure 3:**
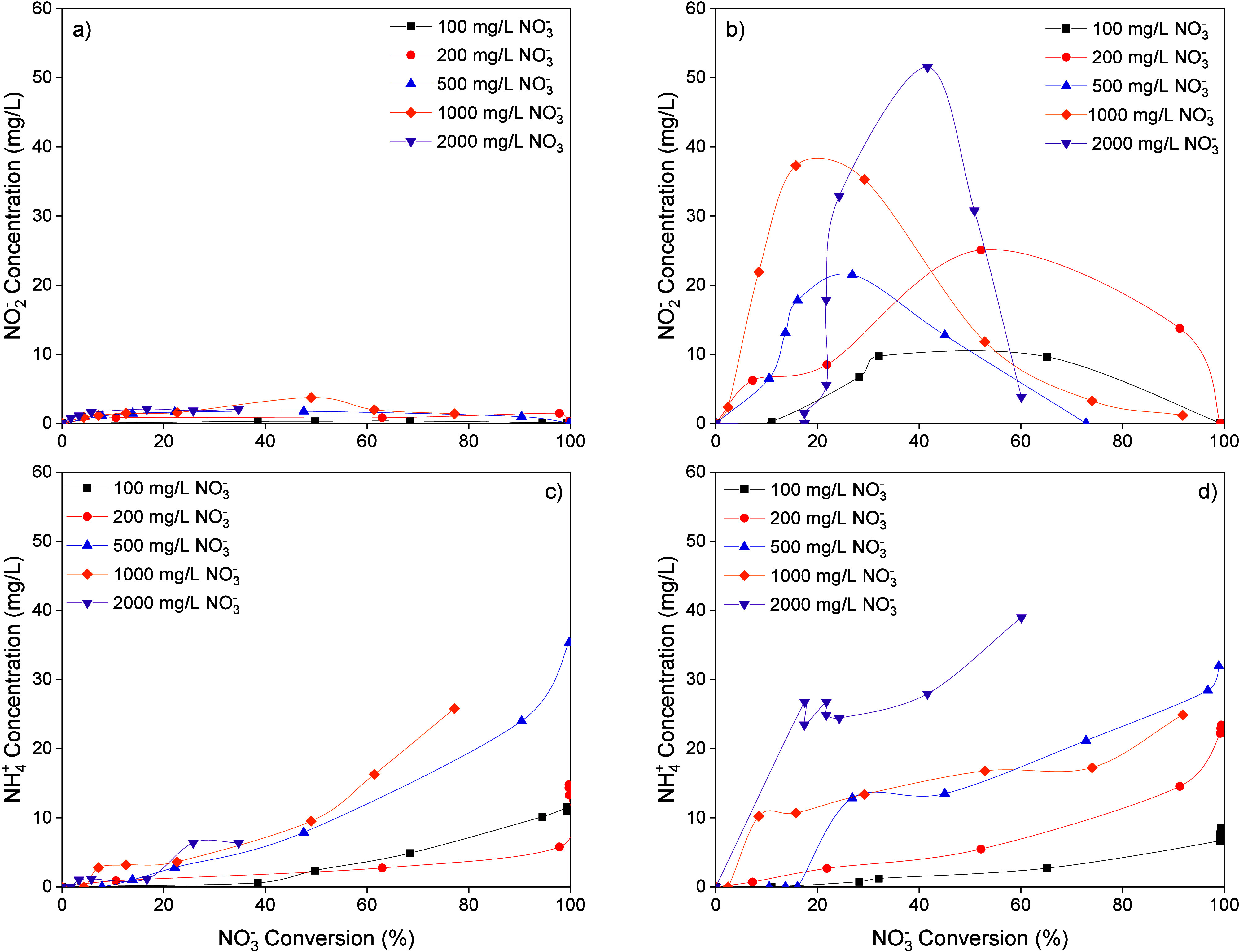
(a) NO_2_^–^ and (c) NH_4_^+^ concentration vs NO_3_^–^ conversion using CO_2_ (50 N mL/min) as the pH control agent, (b) NO_2_^–^ and (d) NH_4_^+^ concentration vs NO_3_^–^ conversion using H_2_SO_4_ (0.05 M) as the pH control agent at different NO_3_^–^ initial concentrations (H_2_ flow: 50 N mL/min, 0.4 g/L catalyst).

### 3.2 Influence of Catalyst Concentration

Tests were carried out with an initial NO_3_^–^ concentration of 1000 mg/L at different catalyst concentrations, using CO_2_ and H_2_SO_4_ for pH control. As the catalyst concentration increased, higher NO_3_^–^ conversion rates were obtained (Figures 4a and 4d), due to the availability of more active sites for NO_3_^–^ reduction. Total NO_3_^–^ conversion was achieved in the reactions using CO_2_ as the pH control agent with catalyst concentrations of 0.8 and 0.93 g/L, at 240 and 180 min of reaction, respectively. For the reactions performed with H_2_SO_4_, similar NO_3_^–^ conversion rates were achieved. Figures 4b, 4c, 4e, and 4f show the evolution of NO_2_^–^ and NH_4_^+^ concentrations for both pH control agents. A maximum in the NO_2_^–^ concentration was observed at different catalyst concentrations for both agents. A lower peak was obtained for the highest catalyst concentration when using CO_2_ for the pH control. However, a higher NO_2_^–^ concentration peak occurred with a catalyst concentration of 0.93 g/L when using H_2_SO_4_, indicating that despite the higher concentration of Pd sites, their availability for NO_2_^–^ reduction was limited due to the presence of SO_4_^2–^ in the reaction medium. The NO_2_^–^ concentration decreased as the NO_3_^–^ concentration dropped, due to slower NO_2_^–^ generation and less competition for NO_3_^–^ reduction. For NO_3_^–^ conversion levels above 99%, complete NO_2_^–^ removal was achieved in all reactions.

**Figure 4:**
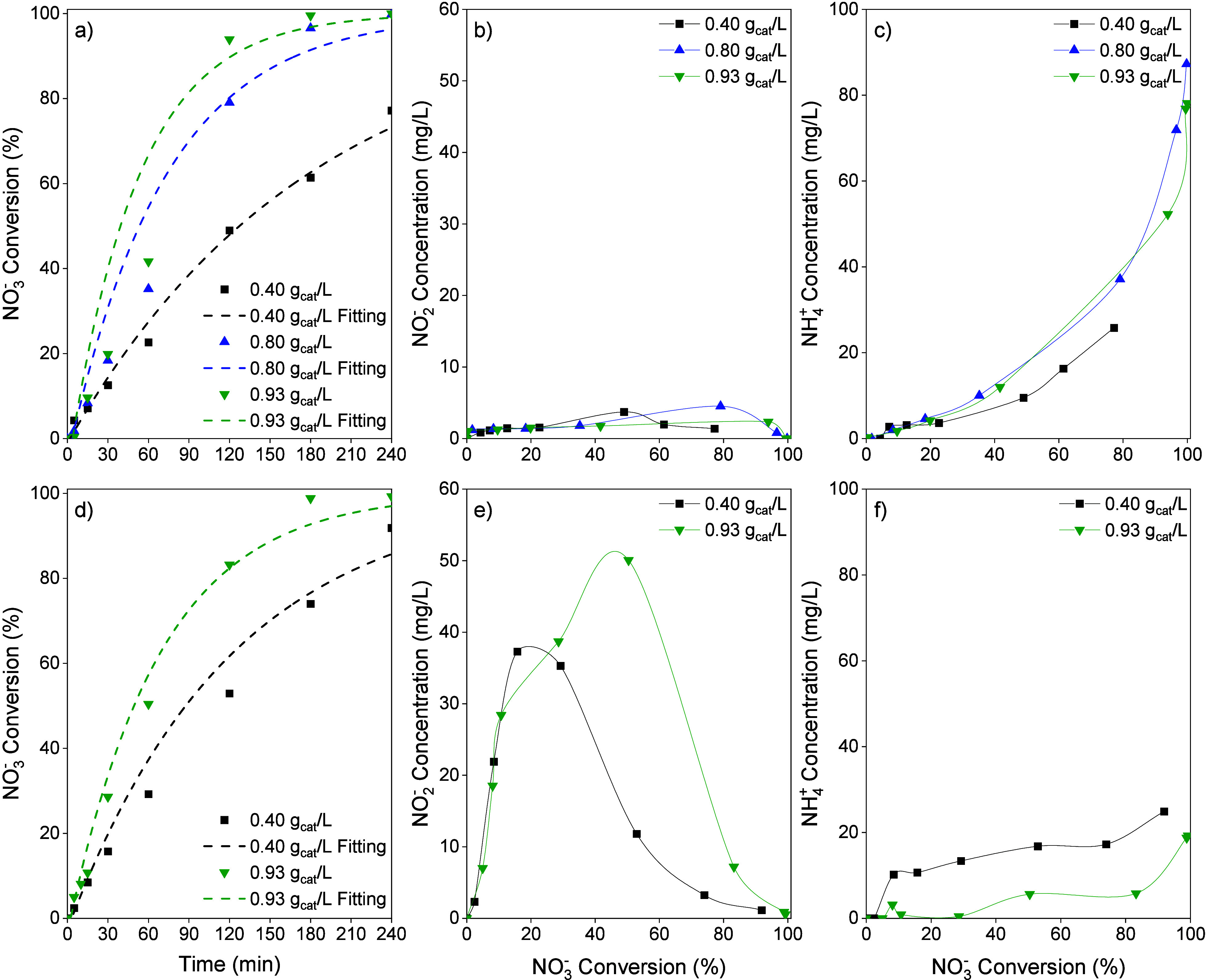
(a) NO_3_^–^ conversion vs reaction time, (b) NO_2_^–^ and (c) NH_4_^+^ concentration vs NO_3_^–^ conversion using CO_2_ (50 N mL/min) as the pH control agent; (d) NO_3_^–^ conversion vs reaction time, (e) NO_2_^–^ and (f) NH_4_^+^ concentration vs NO_3_^–^ conversion using H_2_SO_4_ (0.05 M) as the pH control agent for assays with different catalyst concentrations (H_2_ flow: 50 N mL/min, NO_3_^–^ initial concentration: 1000 mg/L).

Figure 4c shows that the NH_4_^+^ concentration increased with catalyst concentration, especially when the catalyst concentration increased from 0.4 to 0.8 g/L catalyst. These results indicate that the greater availability of active sites increases the hydrogen-to-nitrogen ratio at the active sites, promoting further hydrogenation to NH_4_^+^. In the runs with a catalyst concentration of 0.93 g/L, up to 78 mg/L of NH_4_^+^ were formed after 4 h of reaction, with NO_3_^–^ conversion exceeding 99%. In Figure 4f, the NH_4_^+^ concentration was lower for the run with a higher catalyst concentration using H_2_SO_4_ for pH control, with concentrations reaching 25 and 19 mg/L for catalyst concentrations of 0.4 and 0.93 g/L, respectively. This could be because the previously observed SO_4_^2–^ effect was less pronounced, since a higher availability of active sites due to the higher catalyst concentration would reduce the adsorption competition between nitrogen species and SO_4_^2–^.

In the present work, total NO_3_^–^ removal was obtained with 0.93 g/L catalyst after 180 min of reaction, compared to other works conducted at similar initial NO_3_^–^ concentrations. In a study by Shen et al.,^26^ a 92% conversion was observed in 600 min, using water with an initial NO_3_^–^ concentration of 886 mg/L and a Pd–Cu catalyst supported on an anionic resin (A520E). On the other hand, Marchesini et al.^27^ used a Pd–In catalyst at a concentration of 2.5 g/L and achieved a conversion of 58% after 120 min of reaction, with an initial NO_3_^–^ concentration of 443 mg/L. In the aforementioned studies, NH_4_^+^ concentration was lower than 28 mg/L, as the primary objective was not NH_4_^+^ recovery, unlike in this work. Therefore, exploring alternatives to enhance the NH_4_^+^ production would be beneficial.

### 3.3 NO_3_^–^ Reduction without pH Control

Additional reaction tests were carried out without the addition of a pH control agent, causing the pH to increase from 6.1 to 11.5 by the end of the reaction. Figure 5a shows that total NO_3_^–^ conversion was achieved at 60 min of reaction time in the run without pH control, whereas at the same reaction time, the runs with CO_2_ and H_2_SO_4_ reached only 42 and 50% NO_3_^–^ conversion, respectively. These results contradict the commonly observed effect of OH^–^ in runs without pH control, particularly in studies involving the treatment of polluted drinking water with typical NO_3_^–^ concentrations up to 100 mg/L.^28,29^ It can be inferred that in runs with high NO_3_^–^ concentration, the large amount of HCO_3_^–^ generated or SO_4_^2–^ added has a greater effect on catalytic activity for NO_3_^–^ reduction than the OH^–^ released. However, the activity for NO_2_^–^ reduction was substantially lower in the run without pH control. After nearly complete NO_3_^–^ removal, the NO_2_^–^ concentration in the reaction medium decreased, as it was no longer generated, but the activity remained low, and complete conversion was achieved only after 4 h of reaction. Simultaneously, the level of NH_4_^+^ formation increased, as shown in Figure 5b. In the reaction without pH control, once the NO_2_^–^ concentration started to decrease after 60 min of reaction, the NH_4_^+^ concentration continued to rise from 7 to 49%, indicating that the remaining NO_2_^–^ contributed significantly to NH_4_^+^ formation. Therefore, based on the results, it can be seen that OH^–^ accumulation in the reaction medium affects the activity for NO_2_^–^ conversion and the selectivity to NH_4_^+^ to a greater extent. The increase in NO_2_^–^ concentration suggests that OH^–^ ions produced during the reaction compete for adsorption at Pd active sites, but do not compete for Cu sites, as the NO_3_^–^ reduction rate was not affected. Studies using Pd catalysts for methane combustion have shown that OH^–^ ions can strongly interact with Pd, inhibiting the activity of Pd sites.^30,31^ The most widely accepted mechanism for NO_2_^–^ reduction suggests that it is first reduced to NO on the catalyst surface and then further reduced to NH_4_^+^ or N_2_ through other intermediates.^14^ Herron et al.^32^ analyzed the binding energy of different molecules on Pd sites (Table 2), reporting that OH^–^ ions have a bond strength similar to NO. This could explain the increase in NO_2_^–^ concentration when working without pH control. Furthermore, according to the reaction mechanism, a high hydrogen-to-nitrogen ratio favors the formation of adsorbed intermediate species such as NOH. Herron et al.^32^ also observed a strong binding energy between NOH species and Pd sites, indicating that the formation of these intermediates species is favored, leading to enhanced NH_4_^+^ production.

**Table 2:** Binding Energy on Pd Surface Sites^32^

	Binding Energy (eV)		
	fcc	hcp	Top
NO	–2.07	–2.04	–1.37
NOH	–3.90	–3.78	—
OH	–2.15	–2.02	–1.93

**Figure 5:**
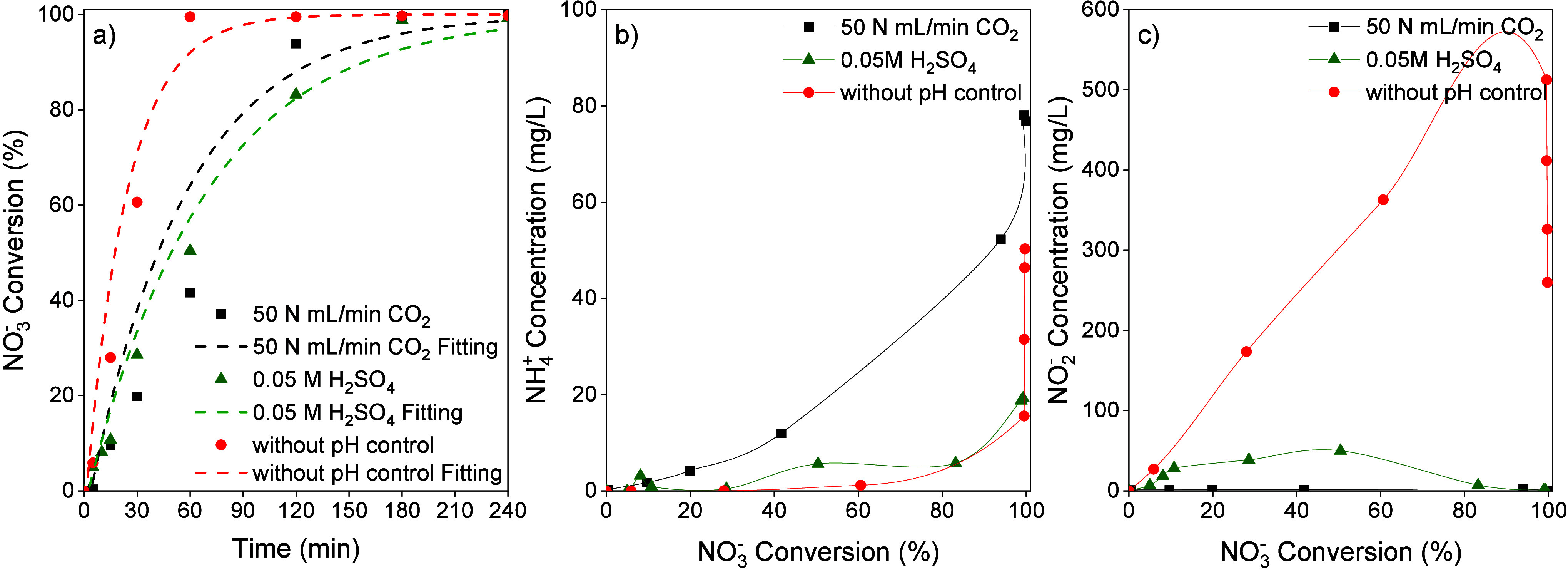
(a) NO_3_^–^ conversion vs reaction time, (b) NH_4_^+^ and (c) NO_2_^–^ concentration vs NO_3_^–^ conversion for assays with different ways to control the pH (H_2_ flow: 50 N mL/min, NO_3_^–^ initial concentration: 1000 mg/L, catalyst concentration: 0.93 g/L).

### 3.4 Synthetic Industrial Wastewater

NO_3_^–^ reduction tests were carried out with S1 and S2 water, using CO_2_ as a pH control agent and without pH control. The results are shown in Figure 6. Total NO_3_^–^ conversion in S1 water was reached at 120 min without pH control and 180 min when CO_2_ was used for pH control. With S2 water, a conversion close to 99% was achieved after 240 min of reaction without pH control. Although complete NO_3_^–^ conversion was observed, the reaction rate decreased compared to the previous tests in reaction media containing only NO_3_^–^. This decrease in catalytic activity may be related to the presence of (NH_4_)_2_SO_4_ in the S1 reaction medium.^20,19^ S2 water presented a similar composition to S1, except for the addition of Cl^–^, Ca^2+^, and NO_2_^–^ in the reaction medium. Despite the addition of salts commonly associated with a loss of catalytic activity, such as Cl^–^,^33^ no noticeable changes in NO_3_^–^ removal were observed. An increase of up to 360 mg/L NO_2_^–^ was observed at 99% of NO_3_^–^ conversion in the run without pH control using S1 water, followed by a decrease in the NO_2_^–^ concentration, accompanied by an increase in the NH_4_^+^ concentration to 75 mg/L, reaching a final NH_4_^+^ concentration of 184 mg/L.

**Figure 6:**
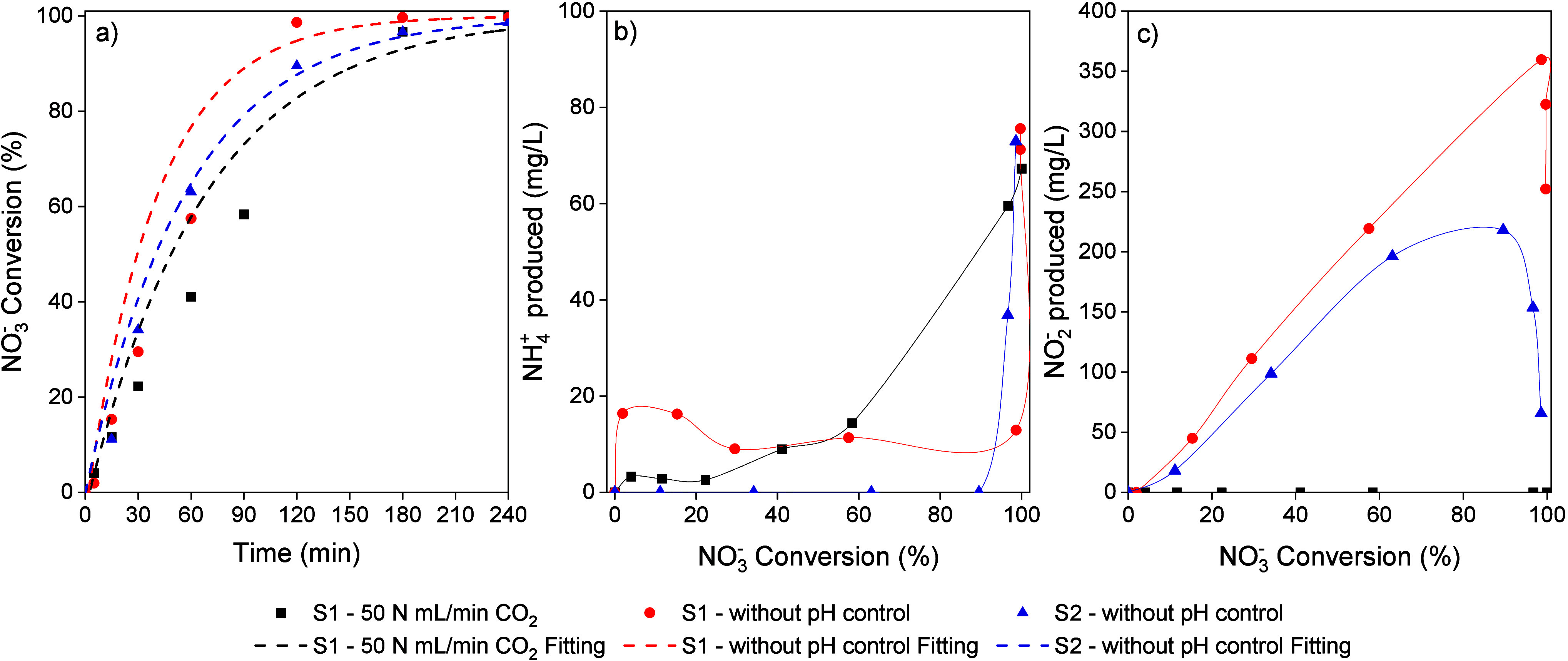
(a) NO_3_^–^ conversion vs reaction time, (b) NH_4_^+^ and (c) NO_2_^–^ produced vs NO_3_^–^ conversion using S1 and S2 as the reaction medium (H_2_ flow: 50 N mL/min, NO_3_^–^ initial concentration: 1000 mg/L, catalyst concentration: 0.93 g/L).

In the run with S1 using CO_2_ as a pH control agent, the NH_4_^+^ concentration increased slightly until a NO_3_^–^ conversion of approximately 60%, producing 67 mg/L NH_4_^+^, with a final NH_4_^+^ concentration of 176 mg/L at the end of the experiment. In the test with S2 water without pH control, an increase in the NO_2_^–^ concentration was also observed, but it was lower than that in S1 water. Up to 217 mg/L NO_2_^–^ was generated, followed by a decrease in the NO_2_^–^ concentration and significant NH_4_^+^ formation for NO_3_^–^ conversion values above 80%. An additional 73 mg/L NH_4_^+^ was produced, reaching a final concentration of 190 mg/L. The additional NH_4_^+^ and NO_2_^–^ generated during the reactions can be seen in Figure S4 (Supporting Information), which shows that the decrease in NO_2_^–^ concentration is accompanied by an increase in NH_4_^+^ in the reaction medium when working without pH control after 120 min of reaction for both waters tested.

### 3.5 Catalyst Reuse

Consecutive reaction runs were carried out in the S2 reaction medium without a pH control. Figure 7a shows the evolution of the NO_3_^–^ conversion over time for successive cycles. After 8 h of reaction, conversion values close to 100% were achieved in all three reaction cycles. However, a gradual decrease in catalytic activity was observed across the cycles, with a noticeable latency at the start of cycles 2 and 3. In Figure 7b, this latency, observed during the first 60 min of the reaction, coincides with a significant increase in the NO_2_^–^ concentration. NO_2_^–^ concentrations of 338 and 429 mg/L were reached at 60 min of reaction for the second and third cycles, respectively. To better understand this effect, a comparison of NO_3_^–^ converted and NO_2_^–^ and NH_4_^+^ produced over time was performed (Figure S5 - Supporting Information). It shows that during the first 60 min of reaction in cycles 2 and 3, a large proportion of NO_3_^–^ was converted into NO_2_^–^. Additionally, a remarkable increase in the level of NO_2_^–^ production was observed across the cycles, peaking at 465 mg/L in cycle 3. In cycle 1, the NH_4_^+^ concentration remained constant until NO_3_^–^ conversion reached approximately 90%. In cycles 2 and 3, NH_4_^+^ generation was delayed and reached very low final concentrations, likely due to reduced activity in the NO_2_^–^ conversion.

**Figure 7:**
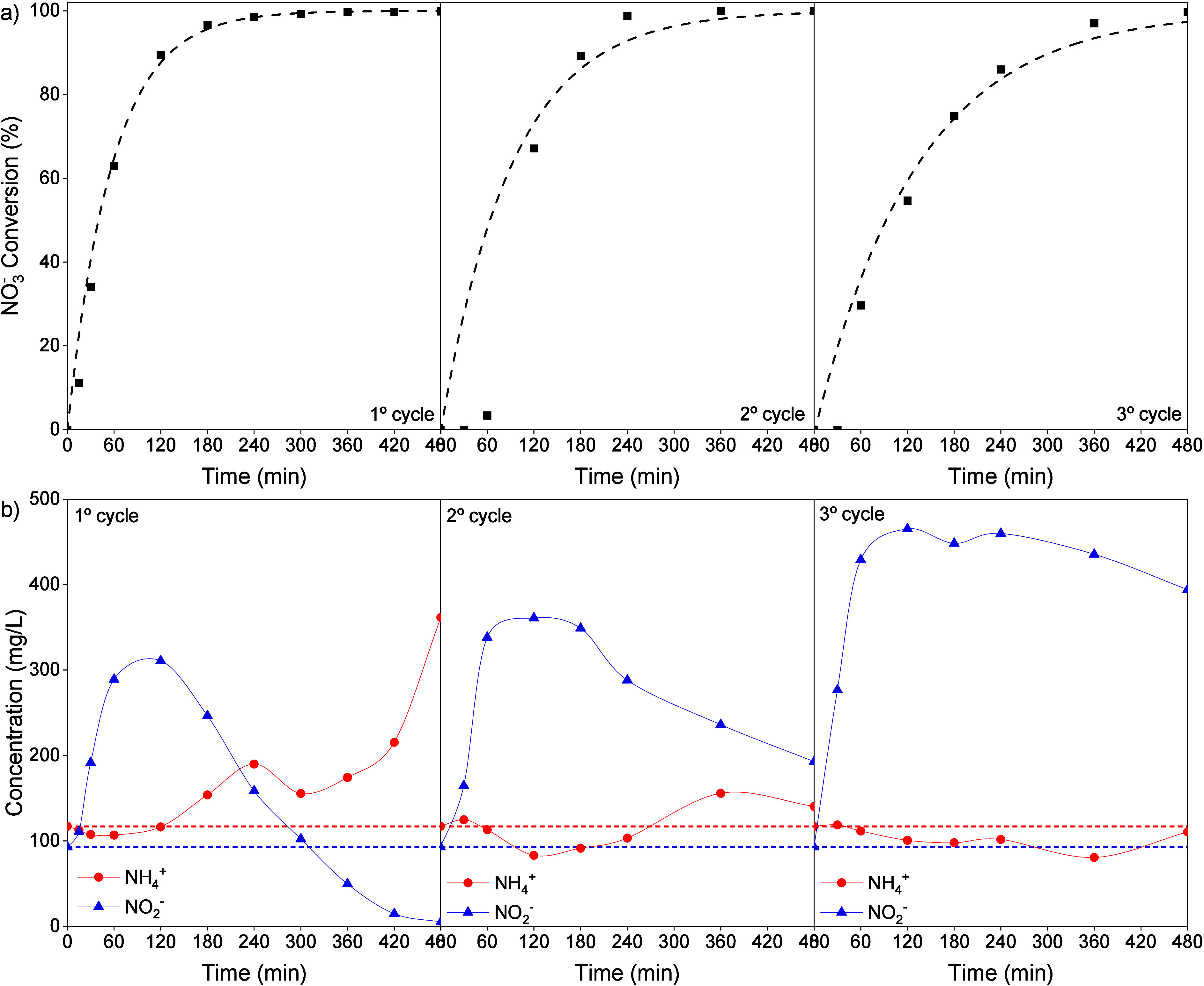
(a) NO_3_^–^ conversion vs reaction time, (b) NH_4_^+^ and NO_2_^–^ concentration vs NO_3_^–^ conversion for successive reaction cycles using S2 as the reaction medium (H_2_ flow: 50 N mL/min, NO_3_^–^ initial concentration: 1000 mg/L, catalyst concentration: 0.93 g/L). Dashed lines: NH_4_^+^ (red) and NO_2_^–^ (blue) initial concentration.

In the literature, the successive use of catalysts for the reduction of NO_3_^–^ in drinking water has been studied. In most cases, a decrease in catalytic activity has also been reported, commonly attributed to catalyst surface poisoning by the presence of salts in the reaction medium or to metallic phase leaching.^34^ In the current work, after cycles 1 and 3, the final aqueous samples were analyzed by TXRF for metal detection, with the results shown in Table 3. For Cu, a small loss in content (0.2–0.3%) was observed, while the leaching of Pd was higher, with losses of 2.1% and 3.8% of its initial content after cycles 1 and 3, respectively. In a study conducted by Bae et al.,^28^ Pd and Cu leaching was analyzed in tests at different pH values, revealing a significant increase in leaching of both metals when the pH exceeded 8. This decrease in Pd content could contribute to a lower level of hydrogenation of the catalyst surface, leading to a decline in catalytic activity and changes in NO_2_^–^ and NH_4_^+^ production.

**Table 3:** Pd and Cu Concentration and Percentage of Metal Lixiviation in Reaction Medium after 1° and 3° Reaction Cycles^a^

		1° cycle	3° cycle
Pd	mg/L	0.7	1.2
	%	2.1	3.8
Cu	mg/L	0.05	0.03
	%	0.3	0.2

a (240 min, [NO_3_^–^]_0_ = 1000 mg/L, H_2_ flow: 50 N mL/min, 0.93 g/L of catalyst).

Fresh and used catalysts after cycles 1 and 3 were analyzed by XPS. The deconvolution of the Pd and Cu peaks is shown in Figure S6 (Supporting Information), and the metal species ratios are summarized in Table 4. After successive reaction cycles, no considerable changes in the proportion of Cu species were observed. Moreover, no peak related to SO_4_^2–^ was observed, in contrast to results in a previous work^18^ where the pH was controlled with CO_2_, despite the high SO_4_^2–^ concentration in the reaction medium. No notable changes in the Pd^0^/Pd^n+^ ratio were observed after cycle 1, but a significantly lower ratio was observed after cycle 3. This could negatively impact catalytic activity, as the literature shows that H_2_ has a higher affinity for adsorption on Pd sites with high coordination.^35^ This is consistent with a lower H_2_ spillover, leading to lower activity in both stages of the reaction.

**Table 4:** Active Phase Composition on the Catalyst Surface for Fresh and Used Pd–Cu/ENS350 Catalysts after 1° and 3° Reaction Cycles Determined by XPS

Catalyst	Pd^0^ (%)	Pd^n+^ (%)	Pd^0^/Pd^n+^	Cu^0^ (%)	Cu^n+^ (%)	Cu^0^/Cu^n+^
Fresh	55.6	44.4	1.3	43.7	56.3	0.8
1° cycle	51.6	48.5	1.1	36.0	64.0	0.6
3° cycle	25.4	74.6	0.3	33.9	66.1	0.5

Furthermore, fresh and used catalysts after cycles 1 and 3 were analyzed by TEM. The EDX maps and nanoparticle size histograms obtained from the images are presented in Figure S7 (Supporting Information). Good dispersion of the metallic phase was observed. The fresh catalyst and that analyzed after cycle 1 showed mean nanoparticle sizes of 3.2 and 2.1 nm, respectively, with a distribution extending up to 6 nm. The catalyst used after cycle 3 exhibited a mean nanoparticle size of 3.0 nm and particles of up to 8 nm.

## 4 Conclusions

The catalyst synthesized in the present work (Pd–Cu/ENS350) demonstrated effective removal of NO_3_^–^ from wastewaters with a high concentration of this pollutant, achieving total conversion after 4 h of reaction using a catalyst concentration of 0.93 g/L. Improved catalytic activity was observed when operating without pH control, allowing for complete NO_3_^–^ removal in a shorter reaction time. High NH_4_^+^ concentrations were obtained, suggesting that catalytic reduction could be a viable technique for NO_3_^–^ removal from industrial wastewater, with the potential for NH_4_^+^ recovery and reuse.

The presence of salts in the simulated wastewater did not significantly affect catalytic activity, as complete NO_3_^–^ elimination was achieved, with the best performance observed in the absence of a pH control. The lack of pH control led to a significant increase in NO_2_^–^ concentration, likely due to OH^–^ ions accumulation on the catalyst surface, which preferentially resulted in hydrogenation to NH_4_^+^, and higher NH_4_^+^ formation at high NO_3_^–^ conversion.

In tests conducted over successive reaction cycles, a gradual decline in the catalytic activity was observed. This decrease in activity was related to changes in the metallic phase, as seen in the characterization of the fresh and used catalysts. Pd leaching was approximately 3.8% after three reaction cycles, and a noticeable decrease in the Pd^0^/Pd^n+^ species ratio occurred, which is likely the primary reason for the loss of activity.

## Supplementary Material



## Notes

Figures with additional information on the experimental setup, concentrations of NO_2_^–^, NO_3_^–^, and NH_4_^+^ during assays, XPS and TEM characterization data, and tables of NH_4_^+^ production and kinetic fitting (PDF)
