# Supplementary material for: Catalytic Reduction of High Concentration Nitrate-Bearing Industrial Wastewater for Ammonium Recovery
Source: ACS ES T Water. 2025 Mar 20;5(4):1595–604. doi: 10.1021/acsestwater.4c00897 (PMC12131187; doi:10.1021/acsestwater.4c00897)
Supplement: Supplementary file 1 [file ew4c00897_si_001.pdf]

Supporting information for

Catalytic reduction of high concentration nitrate-bearing industrial wastewater for ammonium recovery

Dydia Tanisha González[†], José Alberto Baeza*[†,‡], Luisa Calvo[†,‡], Miguel Ángel Gilarranz[†,‡]

[†]Departamento de Ingeniería Química, C/Francisco Tomás y Valiente 7, Universidad Autónoma de Madrid, 28049 Madrid, Spain

[‡]Institute for Advanced Research in Chemical Sciences (IAdChem), Universidad Autónoma de Madrid, 28049 Madrid, Spain

*corresponding author e-mail: josealberto.baeza@uam.es

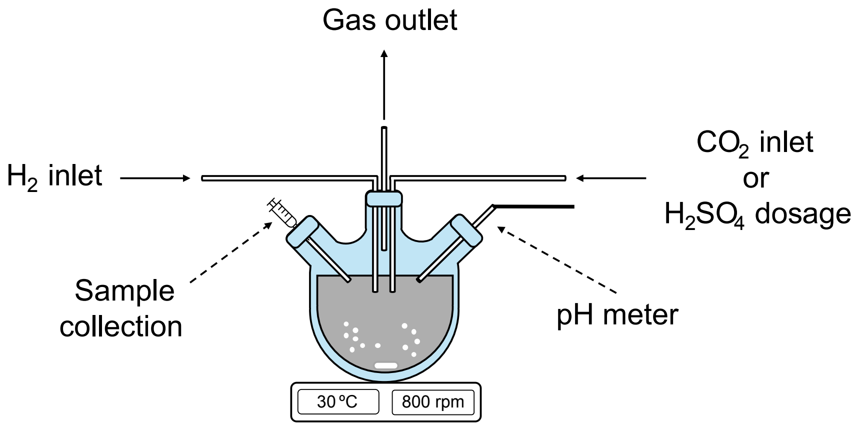

Figure S1. Scheme of the experimental installation.

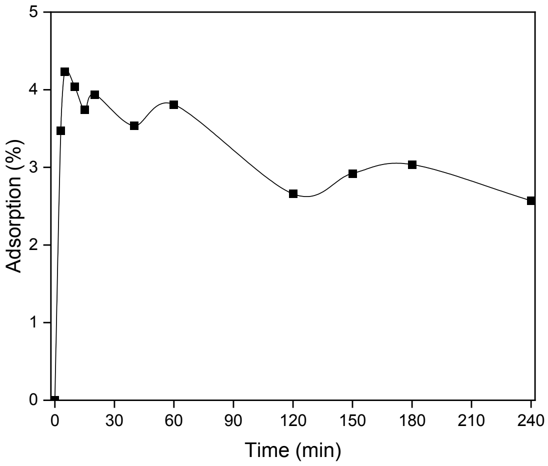

Figure S2. Adsorption test using Pd-Cu/ENS350 catalyst (CO_2: 50 N mL/min, [NO_3^-]: 100 mg/L).

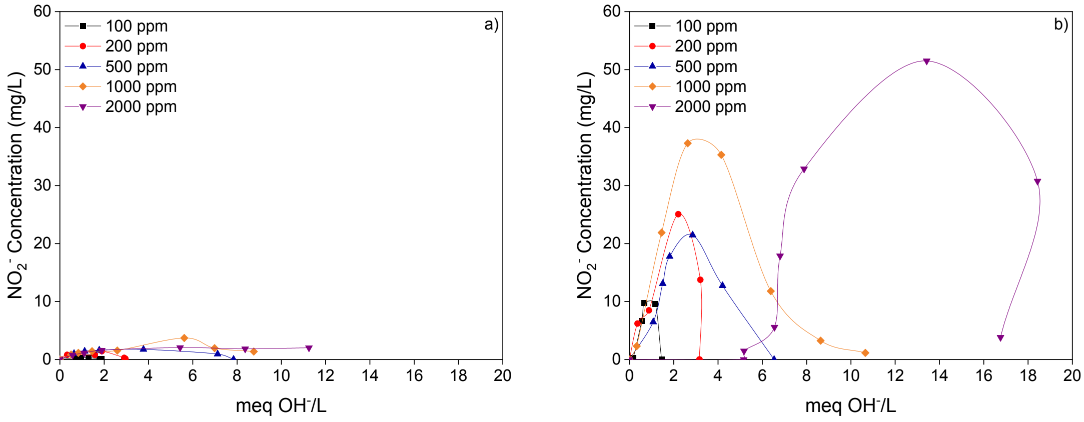

Figure S3. NO_2^- vs OH^- generated from NO_3^- and NO_2^- reduction using (a) CO_2 (50 N mL/min) and (b) H_2SO_4 (0.05 M) as pH control agent at different NO_3^- initial concentration (H_2 flow: 50 N mL/min, 0.4 g/L of catalyst).

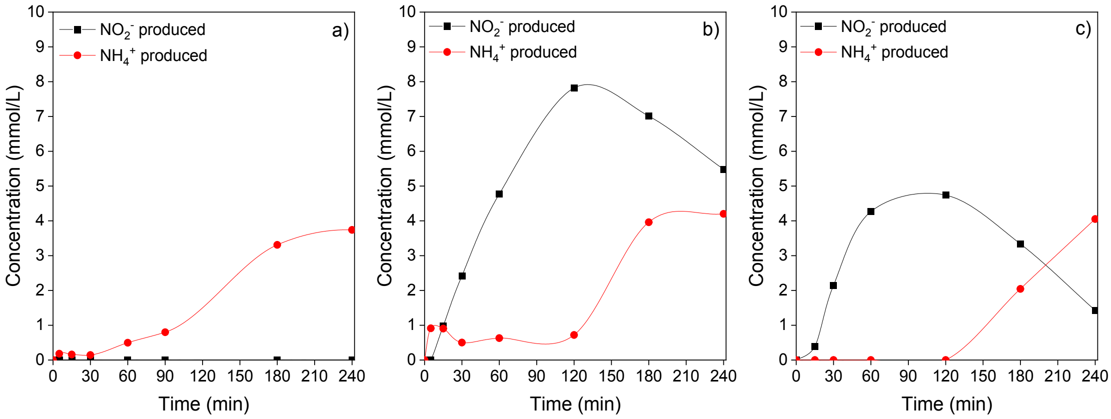

Figure S4. NO_2^- and NH_4^+ generated in mmol/L vs time reaction using (a) S1 water with CO2 as pH control agent, (b) S1 water without pH control and (c) S2 water without pH control (H_2 flow: 50 N mL/min, NO_3^- initial concentration: 1000 mg/L, catalyst concentration: 0.93 g/L).

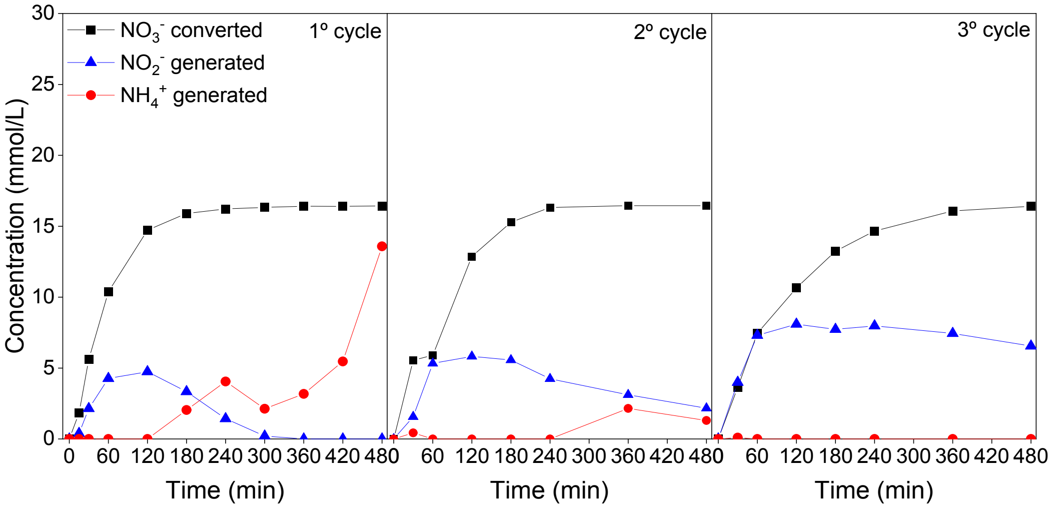

Figure S5. NO_3^- converted, NH_4^+ and NO_2^- generated in mmol/L vs time reaction for successive reaction cycles using S2 as reaction medium (H_2 flow: 50 N mL/min, NO_3^- initial concentration: 1000 mg/L, catalyst concentration: 0.93 g/L).

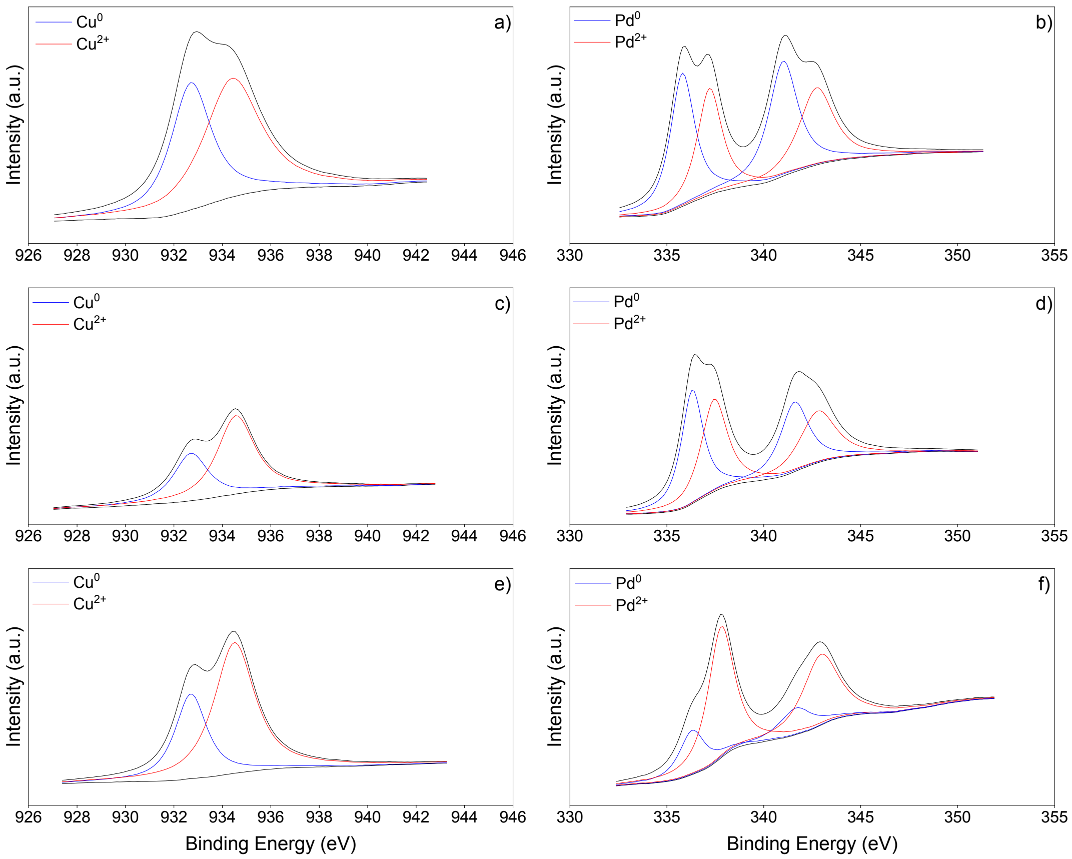

Figure S6. XPS spectra of Cu^0, Cu^{n+}, Pd^0 and Pd^{n+} of Pd-Cu/ENS350 catalyst for the samples (a and b) fresh catalyst, catalyst used after (c and d) 1º cycle and (e and f) 3º cycle.

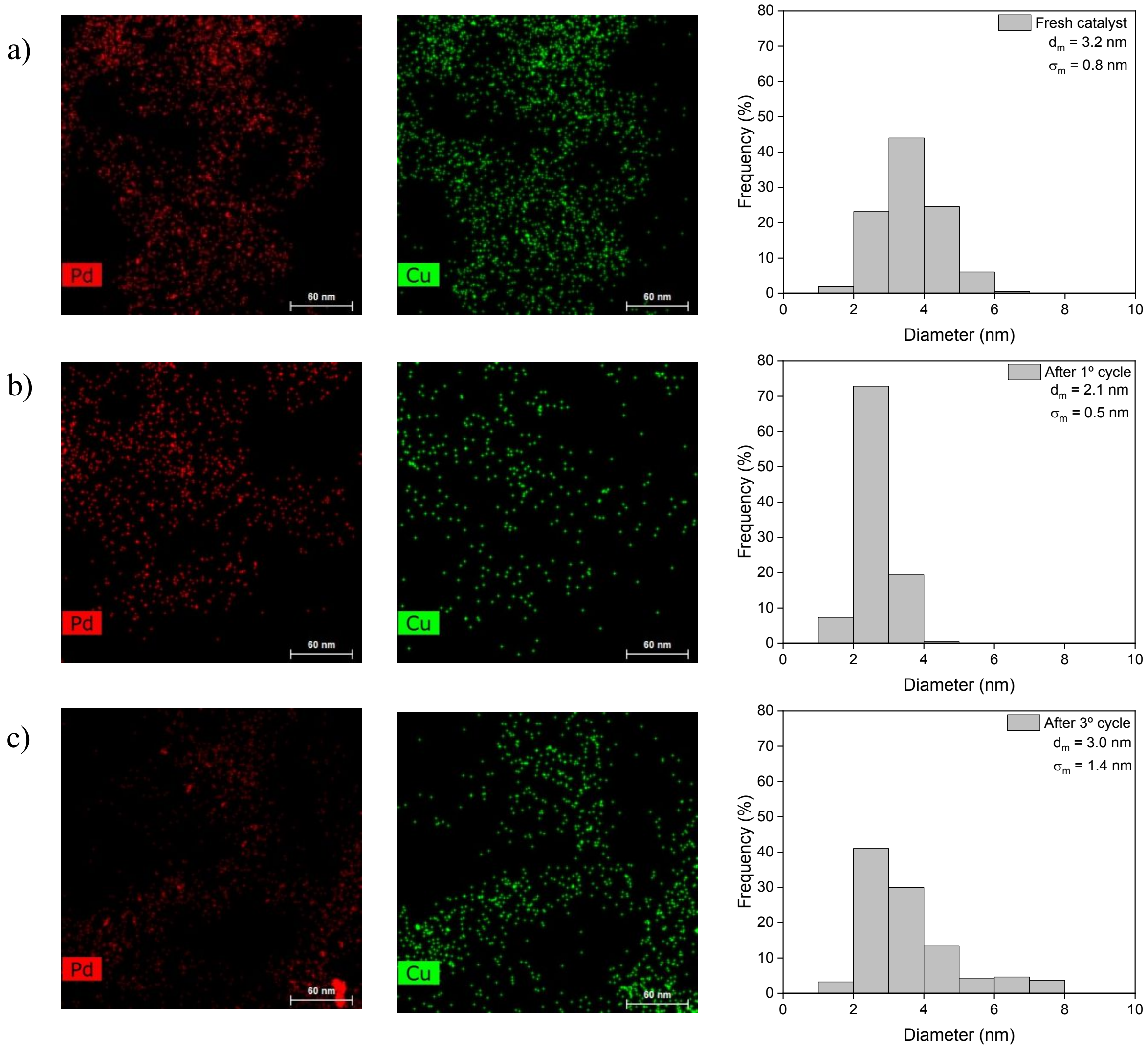

Figure S7. Dispersion maps by EDX characterization and nanoparticle size histograms for (a) fresh catalyst, used catalysts after (b) 1° and (c) 3° cycle reaction by TEM characterization.

Table S1. Production of NH_4^+ production from nitrogen converted.

Reaction conditions		NH_4^+ production (%)
50 N mL/min CO_2	100 mg/L NO_3^-	34.2
	200 mg/L NO_3^-	26.9
	500 mg/L NO_3^-	25.0
	1000 mg/L NO_3^-	16.4
	2000 mg/L NO_3^-	3.2
0.05 M H_2SO_4	100 mg/L NO_3^-	32.9
	200 mg/L NO_3^-	41.1
	500 mg/L NO_3^-	20.2
	1000 mg/L NO_3^-	13.0
	2000 mg/L NO_3^-	13.0
50 N mL/min CO_2 1000 mg/L NO_3^-	0.40 g_{cat}/L	16.4
	0.80 g_{cat}/L	31.8
	0.93 g_{cat}/L	27.3
0.05 M H_2SO_4 1000 mg/L NO_3^-	0.40 g_{cat}/L	13.0
	0.93 g_{cat}/L	9.2
1000 mg/L NO_3^-	50 N mL/min CO_2	27.3
	0.05 M H_2SO_4	9.2
	without pH control	17.4
50 N mL/min CO_2	S1	24.5
without pH control	S1	32.8
	S2	74.1
S2 without pH control	1º cycle	74.1
	2º cycle	7.9
	3º cycle	0

Table S2. Kinetic constants obtained by fitting experimental data to a pseudo-first order kinetic model.

Reaction conditions		k (.10^{-2}) (min^{-1})	R^2	Figure
50 N mL/min CO_2	100 mg/L NO_3^-	4.0	0.970	Figure 2a
	200 mg/L NO_3^-	4.8	0.993	
	500 mg/L NO_3^-	1.8	0.967	
	1000 mg/L NO_3^-	0.6	0.991	
	2000 mg/L NO_3^-	0.2	0.962	
0.05 M H_2SO_4	100 mg/L NO_3^-	4.6	0.990	Figure 2b
	200 mg/L NO_3^-	3.3	0.992	
	500 mg/L NO_3^-	1.3	0.992	
	1000 mg/L NO_3^-	0.8	0.991	
	2000 mg/L NO_3^-	0.3	0.925	
50 N mL/min CO_2 1000 mg/L NO_3^-	0.40 g_{cat}/L	0.6	0.991	Figure 4a
	0.80 g_{cat}/L	1.4	0.991	
	0.93 g_{cat}/L	2.0	0.986	
0.05 M H_2SO_4 1000 mg/L NO_3^-	0.40 g_{cat}/L	0.8	0.991	Figure 4d
	0.93 g_{cat}/L	1.5	0.993	
1000 mg/L NO_3^-	50 N mL/min CO_2	1.8	0.986	Figure 5a
	0.05 M H_2SO_4	1.5	0.993	
	without pH control	4.3	0.987	
50 N mL/min CO_2	S1	1.5	0.994	Figure 6a
without pH control	S1	2.5	0.994	
	S2	1.7	0.999	
S2 without pH control	1º cycle	1.7	0.999	Figure 7a
	2º cycle	1.1	0.989	
	3º cycle	0.8	0.993	
